# Anticoagulant Activity of Cellulose Nanocrystals from Isora Plant Fibers Assembled on Cellulose and SiO_2_ Substrates via a Layer-by-Layer Approach

**DOI:** 10.3390/polym13060939

**Published:** 2021-03-18

**Authors:** Tamilselvan Mohan, Cintil Jose Chirayil, Chandran Nagaraj, Matej Bračič, Tobias Alexander Steindorfer, Igor Krupa, Mariam Al Ali Al Maadeed, Rupert Kargl, Sabu Thomas, Karin Stana Kleinschek

**Affiliations:** 1 Institute for Chemistry and Technology of Biobased Systems (IBioSys), Graz University of Technology, Stremayrgasse 9, 8010 Graz, Austria; tobias.steindorfer@tugraz.at (T.A.S.); rupert.kargl@tugraz.at (R.K.); karin.stanakleinschek@tugraz.at (K.S.K.); 2 Newman College, Thodupuzha, Kerala 685585, India; cintiljose@gmail.com; 3 Ludwig Boltzmann Institute for Lung Vascular Research, Stiftingtalstrasse 24, 8010 Graz, Austria; nagaraj.chandran@lvr.lbg.ac.at; 4 Laboratory for Characterisation and Processing of Polymers, Faculty of Mechanical Engineering, University of Maribor, Smetanova Ulica 17, 2000 Maribor, Slovenia; matej.bracic@um.si; 5 Center for Advanced Materials, Qatar University, Doha 2713, Qatar; igor.krupa@qu.edu.qa (I.K.); m.alali@qu.edu.qa (M.A.A.A.M.); 6 School of Chemical Sciences, Mahatma Gandhi University, Kerala 686560, India; sabuthomas@mgu.ac.in; 7 School of Energy Materials, Mahatma Gandhi University, Kerala 686560, India; 8 International and Inter University Centre for Nanoscience and Nanotechnology, Mahatma Gandhi University, Kerala 686560, India

**Keywords:** cellulose nanocrystals, polyethyleneimine, layer-by-layer, QCM-D, anticoagulant, plasma adsorption, wettability, AFM, profilometer

## Abstract

In this study, we report the isolation of cellulose nanocrystals (CNCs) from Isora plant fibers by sulfuric acid hydrolysis and their assembly on hydrophilic cellulose and silicon-di-oxide (SiO_2_) surfaces via a layer-by-layer (LBL) deposition method. The isolated CNCs were monodispersed and exhibited a length of 200–300 nm and a diameter of 10–20 nm, a negative zetapotential (−34–39 mV) over a wide pH range, and high stability in water at various concentrations. The multi-layered structure, adsorbed mass, conformational changes, and anticoagulant activity of sequentially deposited anionic (sulfated) CNCs and cationic polyethyleneimine (PEI) on the surfaces of cellulose and SiO_2_ by LBL deposition were investigated using a quartz crystal microbalance technique. The organization and surface features (i.e., morphology, thickness, wettability) of CNCs adsorbed on the surfaces of PEI deposited at different ionic strengths (50–300 mM) of sodium chloride were analysed in detail by profilometry layer-thickness, atomic force microscopy and contact angle measurements. Compared to cellulose (control sample), the total coagulation time and plasma deposition were increased and decreased, respectively, for multilayers of PEI/CNCs. This study should provide new possibilities to fabricate and tailor the physicochemical properties of multilayer films from polysaccharide-based nanocrystals for various biomedical applications.

## 1. Introduction

In the last decades, layer-by-layer (LBL) deposition methods have been employed in the field of surface functionalization with nanoscale precision in respect of their chemistry and structure [1,2]. Simplicity offered by LBL method has been widely used to fabricate polyelectrolyte multilayers (PEMs) with different physicochemical properties in various biomedical applications. These comprise their biocompatible or bioactive coatings, biosensors, drug-delivery, cell encapsulation and protein delivery, optically active surfaces, protein-repellent and many others [3]. It can generally be prepared by sequential deposition of polycations and polyanions on various solid substrates [4]. PEMs based on renewable materials, including nanoclays [5], proteins [6], polysaccharides [3,7,8], have been successfully incorporated via electrostatic interactions with charged synthetic polymers [5,9]. Other attractive forces such as hydrophobic interactions and H-bonds may also be involved in the sequential assembly of PEMs [10,11].

In particular, the fabrication of PEM using polysaccharide nanomaterials (e.g., CNCs: cellulose nanocrystals) has attracted great interest for the above mentioned biomedical applications and was first reported by Kotov’s group [12]. CNCs are interesting materials that exhibit several fascinating properties, such as optical activity [13], selective reflection of polarized light [4], strong iridescence [14], anticoagulant activity [15], etc. They are mostly prepared by sulfuric acid hydrolysis of various types of cellulosic materials with a range of aspect ratios (e.g., length: 200 nm–several micrometers, diameter: 5–15 nm) [16,17,18]. However, to best of our knowledge, the current study is the first to describe the isolation of CNCs from Isora plant fibers by sulfuric acid hydrolysis. Another interesting aspect of acid hydrolysed CNCs is that they exhibit negatively charged sulfate groups that confer them an anticoagulant activity, similar to other natural (e.g., heparin) [19] or semi-synthetic polysaccharides such as or cellulose sulfate [8] or sulfo-chitosan [19]. Consequently, the sulfated CNCs can be used to prepare medical coatings or materials that come into contact with blood, such as dialysis tubing, artificial blood vessels, or catheters, etc. [20,21]. As with other anionic polyelectrolytes, CNCs also promote interactions with synthetic or natural cationic polyelectrolytes and thus their incorporation in PEMs. The latter, fabricated from CNCs in combination with synthetic polycations, have been reported in very few studies. For example, CNCs with PAH: poly(allylamine hydrochloride) [22] and poly(DADMAC): polydiallyldimethylammonium chloride were assembled by the sequential deposition of each component. The film properties (e.g., thickness, morphology, roughness, wettability, charges, etc.) and architecture of the PEM assembly were controlled by adjusting the processing conditions such as pH and ionic strength of polyelectrolyte solutions or the type of adsorbing components used for their construction [4,23]. However, no studies have been reported on the assembly of PEMs from the sequential deposition of anionic CNCs and cationic polyethyleneimine (PEI). Especially, a comparative study of LBL assembly of CNC and PEI on two hydrophilic and negatively charged substrates (e.g., cellulose and SiO_2_: silicon-di-oxide) has not been studied in detail, which is the main objective of this present study. Understanding the assembly of multilayers on hydrophilic substrates, in particular on the cellulose platform and potentially from PEI/CNCs, is interesting in terms of developing new cellulose-based composite coatings for medical applications (e.g., anticoagulant surfaces).

Therefore, in this study we isolated CNCs from Isora plant fibers by sulfuric acid hydrolysis treatment. The CNCs were characterized including, their stability in water at different concentrations and particle size in dry and hydrated state using dynamic light scattering and transmission electron microscopy measurements. A quartz crystal microbalance with dissipation (QCM-D) and dip-coating techniques were used for the sequential assembly of PEI and CNCs on hydrophilic cellulose and SiO_2_ substrates. The assembled multilayers were analyzed in detail using various surface sensitive techniques. The coagulation of citrated blood plasma on the self-assembled multilayers were investigated using QCM-D.

## 2. Experimental Section

### 2.1. Materials

Barks of Helicteres isora plant, collected from the tropical region of South Asia [24], were used for the isolation of CNCs. Sodium hydroxide (NaOH), acetic acid (AcOH), sodium hypochlorite (NaClO), sodium chloride (NaCl) and sulfuric acid (H_2_SO_4_) were purchased from Calgon chemicals, Cochin, India. Polyethyleneimine (PEI, branched, high molecular weight) and toluene were purchased from Sigma-Aldrich (Graz, Austria). Trimethylsilyl cellulose (TMSC, DS_TMS_: 2.8, Mw:149,000 g mol^−1^, derived from Avicel PH-101) was purchased from Thüringisches Institut für Textil- und Kunststoff-Forschung e.V. (TITK, Rudolstadt, Germany). Silicon wafers with a thickness of 100 nm and a surface orientation of 100 was purchased from Silchem, (Freiburg, Germany). SiO_2_ coated QCM-D crystals (QSX-301, LOT-Oriel, Germany) were purchased from LOT-Oriel, (Darmstadt, Germany). Ultrapure water from a Millipore water purification system (MA, USA) (resistivity ≥18.2 MΩ cm, pH 6.8) was used for the preparation of all aqueous solutions. All chemicals were used without any purification steps and were of analytical grade.

### 2.2. Cellulose Nanocrystals (CNCs) Preparation

For the preparation of CNCs, we used a slightly modified procedure published in [24]. Step I*:* Isora fibers were chopped into short fibers (size: 0.5–1 cm) and treated with 100 mL of 2 wt.% NaOH in an autoclave. The fibers were treated at 25 psi pressure and at a temperature of 110 °C for 1 h. After depressurization, the fibers were removed from the autoclave and rinsed with ultrapure water several times to remove the traces of NaOH. The steam exploded fibers were then bleached using a mixture of NaOH/acetic acid and sodium hypochlorite solution (1:3, w/w). The bleaching process was repeated 6 times. Afterwards, the fibers were thoroughly washed with ultrapure water and dried at ambient condition. Then the bleached fibers were treated with 10% oxalic acid under a pressure of 25 psi in an autoclave for 15 min. The pressure was released immediately, and the process was repeated eight times. The treated fibers were washed extensively with ultrapure water and dried at ambient condition. Step II: In this step, the bleached fibers from step I were subjected to acid hydrolysis using H_2_SO_4_. Briefly, 270.3 g of ultrapure water was added to 5.9 g of bleached fiber and mixed until a good suspension was obtained. Following this, 529.7 g of H_2_SO_4_ acid (65 wt.%) was slowly added to the suspension and heated to 44 °C for 130 min under constant mechanical stirring. The excess of acid was removed by repeated cycles of water exchange and centrifugation (5000 rpm) for 10 min. Afterwards, the suspension containing CNCs was washed with ultrapure water followed by homogenization using an Ultra-Turax T25 homogenizer (IKA, Staufen, Germany). Finally, the sample was neutralized by the dropwise addition of 1 wt.% NaOH solution to the CNCs suspension.

### 2.3. Inductively Coupled Plasma Mass Spectrometry (ICPMS)

The sulfur content of CNCs was determined using inductively coupled plasma mass spectrometry (ICPMS). Aliquots (~50 mg) of the dried CNCs were weighed to 0.1 mg into 12 mL quartz digestion vessels and 5 mL of HNO_3_ were added. The samples were heated in the UltraClave IV (EMLS, Leutkirch, Germany) using the following program: Step 1: 5 min => 80 °C; step 2: 15 min => 150 °C; step 3: 15 min => 250 °C; step 4: 30 min at 250 °C. For the sulfur determination an Agilent 7500ce at m/z 34 system was used. The instrument was tuned to give 9.0·105 cps at m/z 7 for a 1 µg Li/L, 10·105 cps at m/z 89 for a 1 µg Y/L solution, and 7.5·105 cps at m/z 205 for a 1 µg Tl/L solution. The oxide ratio 156 CeO / 140 Ce was less than 0.013. As internal standard Be at m/z 9 was used. Data acquisition and evaluation were performed with ICPMS Masshunter B.01.01 (Build 123.10Patch 3) software by Agilent (Santa Clara, CA, USA) The sulfur content was determined to be 6.2 g sulfur in 1.0 kg CNCs.

### 2.4. UV−Vis Absorption Spectroscopy

The stability of CNCs in ultrapure water was investigated by UV-Vis spectroscopy. CNCs at different concentrations (0.01–1.0 wt.%) were ultrasonicated for 30 min. The kinetic stability of the samples was determined by transmission measurements at a wavelength of λ = 600 nm (optical path length of 10 mm). The cuvette was capped to avoid a loss of water during the measurements.

### 2.5. Dynamic and Electrophoretic Light Scattering

The nanoparticles’ mean hydrodynamic diameter and polydispersity index (PDI) were determined by dynamic light scattering (DLS) using a Brookhaven Instruments ZetaPlus zeta-potential Analyzer (Holtsville, NY, USA) (wavelength: 674 nm, scattering angle: 90°). Mean particle diameters were approximated as the effective (z -average) diameters. The width of the distribution and the PDO were achieved using the cumulants method, presuming spherical particle shape and log-normal size distribution. The measurements were repeated five times for each sample.

The nanoparticles’ effective zeta-potential [25] was measured by electrophoretic light scattering (ELS) using a Brookhaven Instruments ZetaPlus zeta-potential Analyzer in PALS (wavelength: 674 nm). The effective zeta-potential [25,26] was calculated using the Smoluchowski equation. The measurements were repeated five times for each sample. The samples were diluted with ultrapure water (10 × 10^–3^ wt.% particle content) for both measurements.

### 2.6. Transmission Electron Microscopy (TEM)

Transmission electron microscopy (Philips, WA, USA) (model Philips CM 200) was used to determine the size, shape and morphology of CNCs generated from the isora fibers. A drop of diluted CNCs suspension (0.01 wt.%) was dropped on the surface of a clean copper grid coated with a thin carbon film. As for contrast in TEM, the CNCs were negatively stained in a 2 wt.% solution of uranyl acetate. The sample was dried at ambient temperature before TEM analysis and the measurement was carried out with an accelerating voltage of 80 kV.

### 2.7. Substrate Cleaning and Cellulose Thin Film Preparation

For spin coating of TMSC, Si-wafers and QCM-D Au-coated crystals were used. Si-wafers were cut into pieces of 2 cm × 2 cm, rinsed with ethanol, rinsed with water and immersed into a “piranha” solution (H_2_SO_4_ (98 wt.%)/H_2_O_2_ (30 wt.%), 70:30, v/v) for 15 min. Afterwards, the wafers were rinsed with water, stored in water for at least 15 min and blow-dried with nitrogen gas. QCM-D Au-crystals were soaked into a mixture of H_2_O/H_2_O_2_ (30 wt.%)/NH_4_OH (5:1:1; v/v/v) for 10 min at 70 °C, then immersed in a ‘‘piranha’’ solution for 40 s, and then rinsed with water and finally blown dry with N_2_ gas. For spin coating of TMSC on Au-coated crystals, 50 µl of TMSC solution (1% (w/v, dissolved in toluene, filtered using 5 µm PTFE filter), was deposited onto the static substrate, then rotated for 60 s at a spinning speed of 4000 rpm and an acceleration of 2500 rpm s^−1^. For converting TMSC into pure cellulose, the TMSC coated crystals were placed into polystyrene petri dishes (5 cm in diameter) containing 3 mL of 10 wt.% hydrochloric acid (HCl). The dishes were covered with their cap, and the TMSC films were exposed to vapors of HCl for 15 min. For creating regenerated cellulose coating onto cleaned Si-wafers, the same procedure as mentioned before was used, but 200 µL of TMSC was used for spin coating. A more detailed description concerning the preparation of cellulose thin films using TMSC can be found elsewhere [27,28,29]. The regeneration of cellulose from TMSC was verified by water contact angle, XPS and ATIR-IR measurements as reported elsewhere [27,29,30]. Water contact angles of TMSC and regenerated cellulose films were determined to be 98 ± 1° and 30 ± 2°, respectively. The film thickness of the regenerated cellulose films was 24.0 ± 0.4 nm.

For creating multilayers of PEI/CNCs onto QCM-D SiO_2_ crystals, the “piranha” treatment and cleaning procedure as mentioned above was used.

### 2.8. Sample Preparation for Multilayer Coatings

For the preparation of multilayers on cellulose and SiO_2_ surfaces using QCM-D, PEI and CNCs with a concentration of 0.1 and 0.05 wt.% in ultrapure water (pH 7) were prepared. The ionic strength of the PEI solution was adjusted to 50, 100 and 300 mM with NaCl.

### 2.9. Creation of Multilayers from CNCs and PEI Using QCM-D

A QCM-D instrument (model E4) from Q-Sense, Gothenburg, Sweden was used. The instrument simultaneously measures changes in the resonance frequency (∆f) and energy dissipation (∆D) when the mass of an oscillating piezoelectric crystal changes upon adsorption on the crystal surface. Dissipation refers to the frictional losses that lead to damping of the oscillation depending on the viscoelastic properties of the material. Thus, by measuring the frequency and dissipation it becomes possible to analyse the state of molecular layers bound to the sensor surface during, for example, CNCs or PEI. For a rigid adsorbed layer that is fully coupled to the oscillation of the crystal, Δf is given by the Sauerbrey Equation (1).

(1) $$\Delta m=C\frac{\Delta f_{n}}{n}$$

where *∆f* is the observed frequency shift, C is the Sauerbrey constant (0.177 mg Hz^−1^ m^–2^ for a 5 MHz crystal), n is the overtone number (n = 1, 3, 5, etc.), and *Δm* is the change in mass of the crystal due to the adsorbed layer. The mass of a soft film is not fully coupled to the oscillation and the Sauerbrey relation is not valid since energy is dissipated in the film during the oscillation. The damping (or dissipation) (*D*) is defined as

(2) $$D=\frac{E_{\mathrm{diss}}}{2{\pi E}_{\mathrm{stor}}}$$

where E_diss_ is the energy dissipated, and E_stor_ is the total energy stored in the oscillator during one oscillation cycle. The QCM-D records the change in *D* (*ΔD*) as a function of adsorption time. For the data analysis in this study, the changes in the third overtone‘s frequency and dissipation (*Δf_3_*, *ΔD_3_*) were determined. 

#### 2.9.1. Layer-by-Layer (LBL) Adsorption of PEI and CNCs on SiO_2_ and Cellulose Surface

For the LBL assembly of PEI and CNCs using QCM-D, two types of substrates were used; (i) regenerated cellulose from TMSC and (ii) piranha treated SiO_2_. The cellulose coated Au- or piranha cleaned SiO_2_ crystals were assembled in the QCM-D chamber. In each run, ultrapure water (flow rate = 0.1 mL min^−1^) was pumped into the chamber for 20 min followed by NaCl solutions (50 or 100 or 300 mM) to ensure the stability of the baseline frequency. After establishing a baseline in the respective NaCl solution, PEI solution (0.1%, w/v, dissolved in either 50 or 100 or 300 mM NaCl solution) was introduced into the QCM flow cell for 20 min. Following this, NaCl solutions (either at 50 or 100 or 300 mM) was applied followed by ultrapure water for 10 min. Then, CNCs (0.05%, w/v, in water) were pumped followed by water for 20 min and 10 min, respectively. These procedures (introduction of PEI/NaCl/H_2_O and CNCs/H_2_O) were repeated two more times. In this way, three bilayers were formed. For comparison, the multilayers were also created using PEI but in the absence of electrolyte. All experiments were conducted in continuous flow mode, in which the coated surfaces were constantly exposed to fresh polymer and electrolyte solution or ultrapure water. The temperature was kept at 21 ± 1° C for the duration of the measurement.

#### 2.9.2. Anticoagulant Activity

The LBL assembled PEI/CNCs substrates were tested for their anticoagulant activity by evaluating fibrin clot formation of citrated blood plasma via QCM-D. A regenerated cellulose film was used as a positive control. The substrates (cellulose, bilayer 1 and bilayer 3 of PEI/CNCs fabricated on cellulose at 50 and 300 mM NaCl) were mounted in an open QCM-D cell made of poly(tetrafluoroethylene) for the fibrin clot formation experiments. The open cell allows adding of reactants manually through a round opening during measurements. In this fashion, 100 μL of citrated normal blood plasma (derived from human whole blood and free from cells, ORKE 41, HYPHEN, Biomed, Paris, France), equilibrated to 37 °C, was deposited on the mounted substrate. After 2 min of baseline establishment, coagulation was triggered by adding 100 μL of aqueous 0.025 M CaCl_2_ solution (HYPHEN, Biomed, Paris, France). The experiments were conducted until stable Δ*f* and Δ*D* were obtained, signaling the end of the coagulation process. All experiments were carried out in triplicates at a constant temperature of 37 °C, and their arithmetic mean curves are shown as results. The *Δf_3_* and *ΔD_3_* as a function of time give details about the onset thrombin formation time, fibrin deposition, total coagulation time, and clot density [8,31,32].

#### 2.9.3. Plasma Adsorption

Au-crystals coated with cellulose and multilayers fabricated on cellulose at 300 mM NaCl was assembled in the QCM flow module. The coated films were first equilibrated with water and PBS buffer (pH 7.4) until a stale change in frequency and dissipation was obtained. Then, citrated human blood plasma (derived from human whole blood, free of cells) dissolved in PBS buffer (2 mg mL^−1^, 100% plasma, as recommended by the manufacturer) was pumped for 35 min followed by PBS buffer and water. The measurements were performed in triplicate at 37 °C with a flow rate of 0.1 mL min^−1^.

### 2.10. Profilometry

Layer thickness of the coated films was determined by profilometry using a DEKTAK 150 Stylus Profiler from Veeco (Plainview, New York, NY, USA). The scan length was set to 1000 μm over the time duration of 3 s. The diamond stylus had a radius of 12.5 μm and the force was 3 mg with a resolution of 0.333 μm/sample and a measurement range of 6.5 μm. The profile was set to hills and valleys. Prior to the surface scanning, the coating was scratched to remove the films in order to determine the thickness of the coating using a step-height profile. The thickness was determined at 3 independent positions.

### 2.11. Atomic Force Microscopy (AFM)

Surface topography and roughness parameters of the coated layers ere characterized using a Keysight 7500 AFM multimode scanning probe microscope (Keysight Technologies, Santa Barbara, CA, USA). The images were scanned in tapping mode with silicon cantilevers (ATEC-NC, Nanosensors, Wetzlar, Germany) at an ambient temperature in air (resonance frequency of 210–490 kHz and a force constant of 12–110 N m^−1^). All images were recorded with a resolution of 2048 × 2048 pixels and were processed using the freeware Gwyddion software (Brno, Czech Republic) allowing for the AFM roughness to be calculated as the root mean square (RMS) deviation from the mean height of the topography after leveling of the images by mean plane subtraction and using Equation (1) [33].

(3) $$R_{q}=\sqrt{\frac{1}{N}}\;\sum_{j=1}^{N}r_{j}^{2}$$

## 3. Results and Discussion

### 3.1. Stability, Zetapotential and Size of Cellulose Nanocrystals (CNCs)

**Figure 1:**
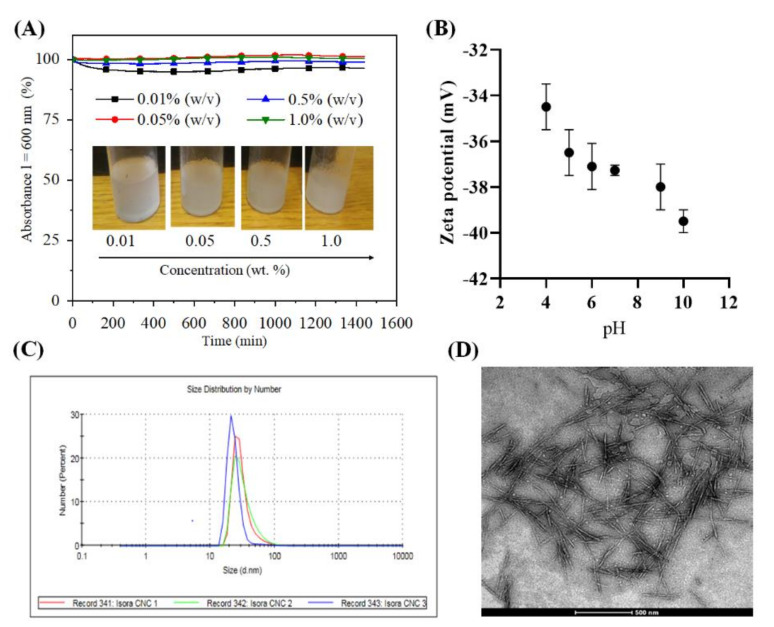
(**A**). Absorbance of Cellulose nanocrystals (CNCs) dispersed in ultrapure water at different concentrations, (**B**) zetapotential, (**C**) particle size distribution (CNC 1: 0 day, CNC2: 1 week, CNC 3: 3 week) and (**D**) Transmission electron microscopy (TEM) image of 0.05 % (w/v) CNCs dispersed in ultrapure water.

We isolated cellulose nanocrystals (CNCs) for the first time from isora plant fibers by sulfuric acid treatment and characterized their stability, charge and morphology in detail using several analytical tools. Figure 1A shows, the observed UV absorbance (λ = 600 nm) of CNCs dispersed in water at different concentrations (0.01–1 wt.%) for 24 h. Absorbance values above 90% were observed for all CNCs concentrations, with a maximum of 100% absorbance for 0.5 and 1.0 wt.% CNCs throughout measurement period. This indicates that the cellulose nanocrystals at all tested concentrations were stable in water over an extended period of time (~24 h) due to electrostatic repulsion. This observation is attributed to the presence of a high density of negatively charged, acidic sulfate ester groups (−OSO_3_^−^ H^+^) (6.2 g/Kg CNCs) on the CNCs surfaces formed by the sulfuric acid hydrolysis treatment [15,34,35,36]. It should be noted that the stability over a period of 24 h should be sufficient for several practical applications. The introduction of sulfate groups on the CNCs surfaces can be successfully followed by zeta potential measurements. Figure 1B shows the zeta potential values of the CNCs (0.05 wt.%, dispersed in water) from acidic to alkaline pH. As anticipated, a highly negative zeta potential (−34–39 mV) was observed over a wide pH range (4–10), which agrees well with the results obtained for the CNCs prepared from various other cellulose sources, including microcrystalline cellulose, jackfruit peel, bacterial cellulose, cotton linters and so on [15,37].

The hydrodynamic diameter of CNCs (0.05 wt.%, dispersed in water) stored at different time intervals (CNC1: 0 h, CNC2: 1 weak, and CNC3: 3 week) is shown in Figure 1C. From the number dependent size distribution curve (Figure 1C), it can be noted that the freshly prepared CNCs had a hydrodynamic diameter of 30 nm; the latter values correlate well with those reported for CNC obtained from microcrystalline cellulose, tunicate, cotton filter paper [15,38,39]. The remaining 70–75% had diameters ranging from 15 nm to 90 nm. However, these particles accounted for less than 1% of all particles in both cases. Most of the 70–75% particles had a diameter of 25–35%, which deviated only ± 5% from the mean value of 30%. This is indicative of a monodisperse particle distribution. We also measured the particle size of the CNCs stored for 1 and 3 weaks, as the stability and storage of nanomaterials over time is a serious problem for various medical applications. The results showed that the particle size of the CNCs was not affected by the storage time, indicating that the CNCs are very stable and no aggregation or flocculation in water was observed. Such stable dispersions, which do not require additional stabilizers, and negatively charged nanoparticles are very suitable for QCM-D measurements, and for introducing anticoagulant activity or surface modification via electrostatic interactions [15]. Figure 1D shows the size and shape of the vacuum dried CNCs. Acid hydrolysis of Isora fibers resulted in the formation of elongated nanocrystals with high aspect ratio, which are also commonly observed for CNCs isolated from other cellulose sources, including microcrystalline cellulose, sisal, cotton, etc. [14]. The TEM image showed CNCs with an average diameter of 10 to 20 nm and length of 200–300 nm. The values obtained correlated well with the literature values of cellulose nanocrystals isolated from various sources such as cotton (*l* =100–300 nm), cotton linters (*l* = 25–500 nm), hard wood (*l* = 140–140 nm), microcrystalline cellulose (*l* = 35–300 nm), bacterial cellulose (*l* = 100–1000), sisal (*l* = 100–500 nm), ramie (*l* = 150–250 nm), tunicate (*l* = 100–1000 nm), etc. [14,15,35]

### 3.2. Multilayer Formation

**Figure 2:**
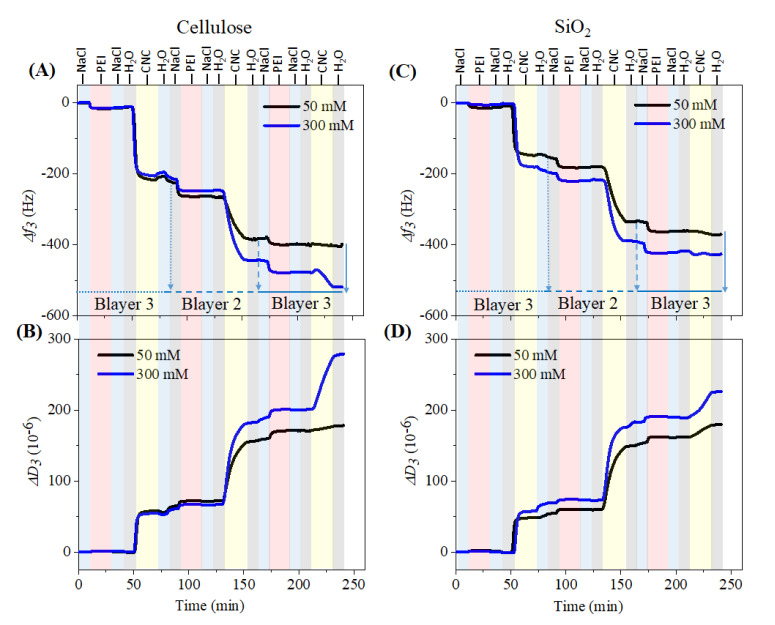
QCM-D change in frequency and dissipation for the sequential adsorption of polyethylenimine (PEI) and CNCs on SiO_2_ (**A**,**B**) and cellulose (**C**,**D**) surface.

Figure 2 shows the changes in QCM-D frequency and dissipation for layer-by-layer (LBL) adsorption of PEI (0.1 wt.%) and CNCs (0.05 wt.%) on cellulose and SiO_2_ surfaces at neutral pH. We used cationic PEI as an anchoring layer for anionic CNCs, since the immobilization or adsorption of CNCs directly (without PEI) on the cellulose and SiO_2_ surfaces was not possible (data not shown). For LBL assembly of PEI/CNCs on both surfaces, we varied only the ionic strength of the PEI solution from 50 to 30 mM with NaCl but not of the CNCs. This was done to understand the adsorption kinetics of PEI on the base layer (i.e., cellulose and SiO_2_) and on the adsorbed CNC layers, and its influence on the deposition rate of the CNCs. A total of three bilayers of PEI/CNCs were created. The LBL assembly of PEI and CNCs on the cellulose surface is shown in Figure 2A,B, respectively. For clarity we have not included the results obtained with 100 mM NaCl. Application of the first PEI solution at 50 mM ionic strength resulted in an irreversibly adsorbed layer (Δf_3_: –16 ± 0.4 Hz) on the cellulose surface, as reflected by a sharp decrease in the frequency, which did not change considerably (Δf_3_: –11 ± 0.2 Hz) after rinsing with NaCl and ultrapure water. This may be attributed to an elevated electrostatic interaction between strongly and positively charged PEI (zetapotential: +35–40 Mv at pH 7–7.4) [40,41] and weakly and negatively charged cellulose surface (zetapotential: −18 to −20 mV at pH 7–7.4) [42]. However, the adsorbed PEI molecules, were in a rigid conformation i.e., they were tightly bound to the surface, as shown by the dissipation change before (*ΔD_3_*: 1.1 ± 0.1 × 10^–6^) and after rinsing (*ΔD_3_*: 0.3 ± 0.02 × 10^–6^). Similar results were obtained for the same PEI solution when adsorbed on a strongly and negatively charged SiO_2_ surface (zetapotential: −80 to −85 mV at pH 7–7.4) [30,32]. The final change in frequency and dissipation in this case was −11 ± 2 Hz (Figure 2C) and 0.2 ± 0.01 × 10^–6^ (Figure 2D), respectively. These results suggest that PEI was adsorbed on both charged surfaces in a similar conformation and the adsorption was strongly dominated by electrostatic interaction [3,15,40]. Further increasing the ionic strength of the PEI solution to 300 mM did not result in any significant adsorption (change in frequency and dissipation) on either surface. These results indicate that the negative charges or binding sites of cellulose and SiO_2_ were sufficiently covered or saturated with positively charged PEI at lower ionic strength. Higher ionic strength led to maximum screening of the charges of PEI (in solution) and cellulose/SiO_2_ surfaces (at the interfaces) and therefore no increased deposition of PEI was observed.

In general, a decrease in frequency (*Δf_3_*) and an increase in dissipation (*ΔD_3_*) were observed when the number of adsorption steps increased. The introduction of PEI in each bilayer resulted in a positive shift in dissipation and a negative shift in frequency, indicating a strong electrostatic interaction of the solid, negatively charged CNCs with positively charged PEI. This interaction is slightly weaker in bilayer 3 where less PEI was seen, which in turn resulted in lower deposition of CNCs, indicating that the electrostatic interaction between PEI and CNCs is not stronger in the bilayer 3 than other two bilayers. This behavior was seen for both cellulose and SiO_2_ surfaces. Moreover, the introduction of PEI solutions showed a less swollen layer, reflected by a smaller increase in dissipation compared to CNCs immobilized layer where several-fold increase in dissipation was noticed. This implies that the PEI was strongly and tightly bound to the CNCs with less amount of incorporated water molecules.

This trend of frequency decrease and dissipation increase was more pronounced at higher ionic strength (300 mM). The maximum adsorption of polymers was observed only up to bilayer 2 compared to bilayer 3. Interestingly, a maximum decrease in frequency was observed during adsorption of the first CNCs layer onto the previously adsorbed PEI layer, even though the differences in frequency change were very negligible for the first PEI layer created with increased ionic strength of NaCl. This behavior was even more pronounced for cellulose. Rinsing with water removed the loosely adsorbed CNCs but most of the adsorbed materials remained on the surface as indicated by a slight increase in frequency. This indicates that a strong electrostatic interaction occurred between the cationic PEI and the negatively charged sulfated CNCs, thus the CNCs was irreversibly attached to the surface. Further rinsing with NaCl (following water) also did not remove the adsorbed CNCs, which was also noticed in all three bilayers, indicating that the adsorbed species of CNCs and PEI were strongly attached to each other between and withing the layers not only by electrostatic interactions but also by forces such as H-bonding, hydrophobic interactions, etc. Since the adsorbed layers were not removed by rinsing with either water or NaCl, it can be concluded that the formed multilayers were highly stable and have a huge potential for use in several biomedical applications. The decrease in frequency during adsorption of CNCs in each bilayer was higher compared to PEI and it was more pronounced as the ionic strength of PEI solutions increased. Such behavior is well known and has been confirmed by several authors for the sequential adsorption of CNCs on the oppositely charged polyelectrolyte layers [15,23,35]. The sequential adsorption of PEI and CNCs can also be followed by the change in dissipation. The latter was proportionally increased during each adsorption step. The maximum increase in dissipation was observed during CNCs adsorption compared to PEI. This indicates that the CNCs adsorbed in a looser conformation and less tightly bound to the underlying layer and incorporated with the maximum amount of water than the PEI.

**Figure 3:**
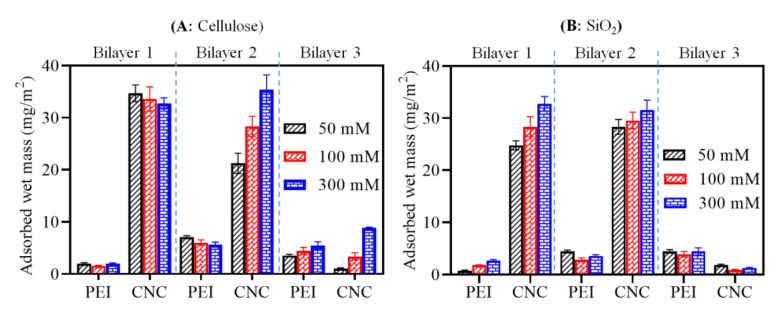
Comparison of adsorbed mass (wet) for the adsorption of PEI and CNCs on the surfaces cellulose (**A**) and SiO_2_ (**B**) as a function of ionic strength of PEI solution.

The final adsorbed wet mass (calculated using Equation (1)) after adsorption of each PEI and CNC layer on cellulose and SiO_2_ is displayed in Figure 3. While no major increase in adsorbed mass (between 1.6 and 1.9 mg/m^2^) was seen for cellulose (A), adsorption of the first PEI layer resulted in an increased mass (50 mM NaCl: 0.8 mg m^−2^, 100 mM NaCl: mg m^−2^ 1.8, 300 mM NaCl**:** 2.7 mg m^−2^) on the SiO_2_ surface (B) as a function of the ionic strength of the PEI solution. This may be due to the higher density of negative charges of SiO_2_ surface compared to cellulose [30,42,43]. Similar to PEI adsorption, the mass of CNCs in bilayer 1 increased on the SiO_2_ surface as a function of the ionic strength of the PEI solution, but not on cellulose, where maximum deposition of mass was already reached at 50 mM NaCl of PEI. This can be related to the presence of higher amounts of PEI on SiO_2_, which caused increased deposition of CNCs due to stronger electrostatic interactions between them. For bilayer 1 on cellulose, slightly more mass (32–35 mg m^−2^) was adsorbed than on SiO_2_ (24–30 mg m^−2^) at all NaCl concentrations. This observed value of both cellulose and SiO_2_ surfaces is comparable to the adsorbed mass (ca. 30 mg m^−2^) of CNCs in bilayer 1 of PEI/CNCs (CNCs isolated from microcrystalline cellulose) in the study by Ehmann et al. [15]. More mass was deposited on cellulose (21–35 mg m^−2^) than on SiO_2_ (28–31 mg m^−2^) for bilayer 2. An explanation may be that the higher density or amount of adsorbed PEI in bilayer 2 caused more adsorption of CNCs via electrostatic interactions. In particular, a gradual increase in the mass of CNCs on the cellulose in bilayer 2 was observed as the concentration of NaCl increased. Interestingly, less PEI and CNCs were adsorbed in the case of bilayer 3. This suggests that with increasing adsorption steps, the accessibility of the remaining free anionic or cationic charges of the CNCs or PEI in the previously adsorbed layers may also be reduced due to steric hindrance or lack of free binding sites on the previously adsorbed CNCs or PEI layer. This steric hindrance and lower electrostatic attraction would result in lower adsorption as the maximum adsorption is approached. The total adsorbed mass observed for the third bilayer on the cellulose surface (1–8 mg m^−2^) was slightly higher than on SiO_2_ (1.2–1.7 mg m^−2^). It should be noted that the calculated total mass was stemming from the combination of polymer, electrolyte and incorporated water molecules in the adsorbed layer.

### 3.3. Surface Morphology, Roughness, Wettability and Layer Thickness 

**Figure 4:**
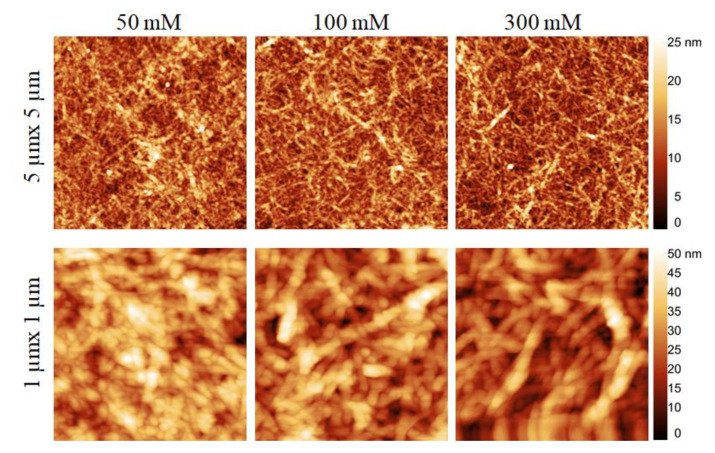
Atomic force microscopy (AFM) height images (**top**: 5 µm × 5 µm, **bottom**: 1 µm × 1 µm) of CNCs coating in bilayer 3 created on cellulose surface as a function of ionic strength of PEI solution.

**Figure 5:**
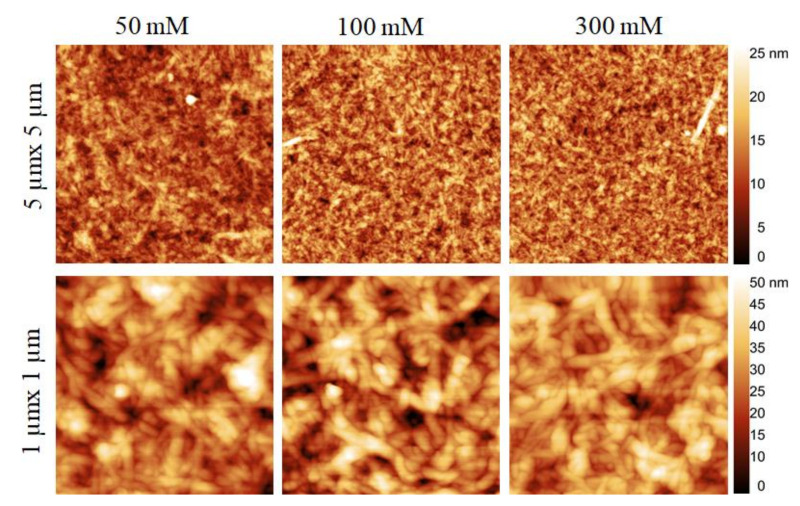
AFM height images (**top**: 5 µm × 5 µm, **bottom**: 1 µm × 1 µm) of of CNCs coating in bilayer 3 created on SiO_2_ surface as a function of ionic strength of PEI solution.

**Figure 6:**
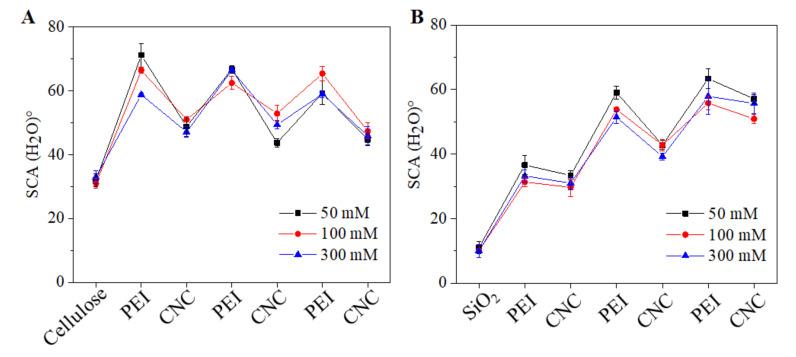
Static water contacts (SCA(H_2_O)°) of the sequentially adsorbed PEI (at 50–300 mM NaCl) and CNCs on cellulose (**A**) and SiO_2_ (**B**).

**Figure 7:**
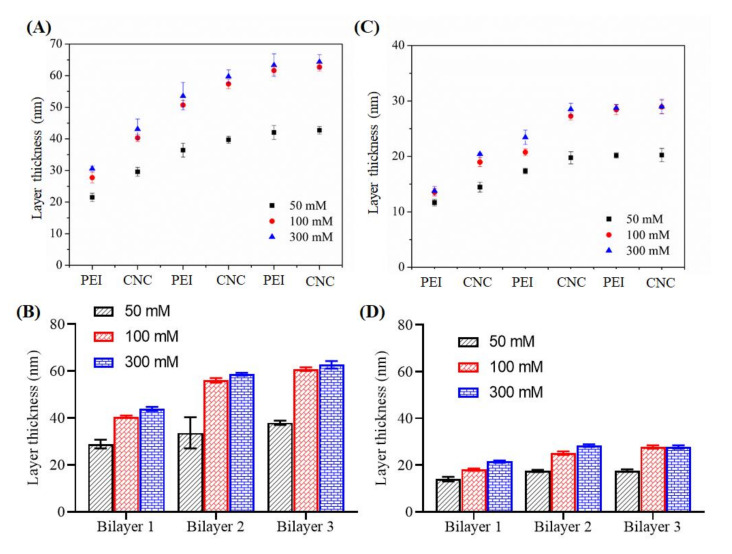
Layer thickness of the sequentially adsorbed PEI (at 50–300 mM NaCl) and CNCs on cellulose (**A**,**B**) and SiO_2_ (**C**,**D**) surfaces.

We analyzed the surface properties (see Figure 4, Figure 5, Figure 6 and Figure 7) of neat cellulose and SiO_2_ surfaces before and after adsorption with multilayers of PEI and CNCs. The surface morphology of neat cellulose and SiO_2_ coated with PEI layer at different ionic strengths of NaCl is shown in Figure S1 (see Supplementary Materials). The morphology and roughness of the neat surfaces were changed after coating with the first PEI layer at different ionic strengths of NaCl (Figure S1 and Table 1). The PEI coated cellulose surface showed higher roughness values (RMS: 6.1–6.7 nm) than the neat cellulose (RMS: 0.9 nm). Compared to SiO_2_, the PEI coated cellulose surfaces showed higher roughness values, indicating that more mass of PEI was adsorbed on the cellulose. This supports the results of QCM-D, which showed increased adsorbed mass (Figure 2 and Figure 3) of PEI on for cellulose. 

Figure 4 and Figure 5 show the surface morphology of CNCs in bilayer 3 coated on cellulose and SiO_2_ at different ionic strength of NaCl. With increasing ionic strengths of NaCl from 50 to 300 mM, the cellulose surfaces (Figure 4) were not only completely covered but also more organized with elongated CNCs. While the surfaces were populated with a higher density of CNCs, the latter were more agglomerated at lower ionic strength (50 mM) of NaCl than at higher NaCl concentration. This was also confirmed by the surface roughness values, which were slightly higher at 50 mM NaCl (RMS: 9.2 nm) compared to 100 (RMS: 8.2 nm) and 300 mM NaCl (8.1 nm). Similar to cellulose, the SiO_2_ surface (Figure 5) was uniformly covered with the adsorbed CNCs. The uniformity of the coated surface increased with increasing NaCl concentration. Also, in this case, the CNCs were adsorbed in the form of agglomerates. The values of surface roughness of the coated surfaces were lower compared to those found for cellulose, confirming a lower deposition of CNC deposition on SiO_2_, which is in good correlation with the QCM-D data (see Figure 3).

The static water contact (SCA(H_2_O) values of neat and coated surfaces are shown in Figure 6. It was expected that the deposition of PEI and CNCs would change the wettability of both neat cellulose and SiO_2_ surfaces. This was verified by the SCA(H_2_O) values of the coated surfaces. While the neat cellulose and SiO_2_ showed SCA(H_2_O) values of 32 ± 2° and 9 ± 1°, respectively. The LBL coated PEI and CNCs exhibited alternatively changes in SCA(H_2_O). This confirmed the sequential deposition of oppositely charged PEI and CNCs on both surfaces (cellulose and SiO_2_). In general, the outer surface of the CNC layer in each bilayer showed reduced SCA(H_2_O) = 30° to 50 ° values compared to PEI. This was also seen for SiO_2_ surfaces and at all ionic strengths of NaCl. The reduced SCA(H_2_O) indicates that the CNCs are more hydrophilic than PEI. In general, for CNCs coated surfaces, SCA(H_2_O) values ranging from 30 to 60° and with moderate hydrophilic character have been reported by several authors [15,44,45]. Overall, lower SCA(H_2_O) values were observed for strongly and negatively charged SiO_2_ surfaces compared to weakly negatively charged cellulose surfaces.

We also measured the layer thickness of each coating using profilometry and the results are shown in Figure 7. The latter showed that the layer thickness increased linearly with increasing adsorption steps of PEI and CNCs. Increase of layer thickness was noted up to bilayer 2 and did not change significantly thereafter. Compared to 50 and 100 mM NaCl, no major increase in layer thickness was observed for adsorption with 300 mM NaCl. As expected, the maximum growth of layer thickness was observed for cellulose surface (30–60 nm) compared to SiO_2_ (18–24 nm). These results correlate well with the QCM-D mass, where the latter technique showed more deposition of polymers on the cellulose than on the SiO_2_ surface at all ionic strengths of the PEI solution (see Figure 3).

### 3.4. Anticoagulant Activity and Plasma Adsorption

**Figure 8:**
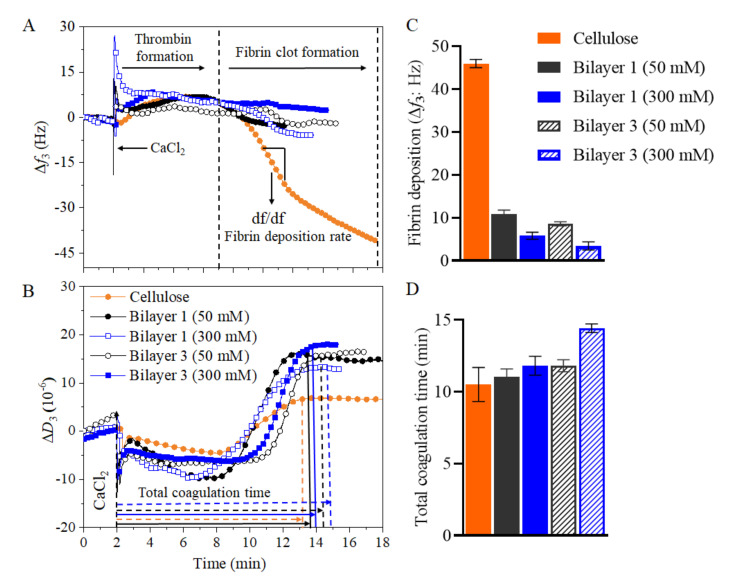
Fibrin clot formation by QCM-D on LBL assembled PEI/CNCs at 50 mM (full symbol) and 300 mM (empty/patterned symbol). Neat PEI and cellulose film as reference materials are shown as well. (**A**) Frequency (*Δf*_3_) and (**B**) dissipation (*ΔD*_3_) changes during coagulation process, (**C**) total fibrin deposition, and (**D**) total coagulation time.

As shown in Figure 8A, when the coagulation cascade was initiated (addition of CaCl_2_ at 2 min) the Δ*f*_3_ slightly increased subsequently followed by a significant drop. The decrease of Δ*f*_3_ reflects the formation of fibrin fibers, which form a clot and deposit on the surface. Later, the *Δf_3_* stabilizes as the coagulation process was finished at this point, total coagulation time can be determined. Nevertheless, the stabilization of *Δf*_3_ was often difficult to determine and the stabilization of *ΔD*_3_ (Figure 8B) was used as it was much pronounced. The *ΔD*_3_ change during clot formation followed a similar trend than Δ*f*_3_. After initiation of the coagulation cascade, the *ΔD*_3_ remained rather stable and starts to increase after the fibrin clot was formed. This reflects the increase in elasticity of the liquid above the quartz crystal to a point of high viscoelasticity, which was dampening the oscillation. After the coagulation process ends, the *ΔD*_3_ recordings were stable as no further changes in elasticity were countered. From Figure 8A and Figure 8C the significant differences in fibrin deposition can be observed on the selected substrate surfaces. For this study, we performed coagulation experiments using citrated blood plasma on multilayers built on cellulose surface rather on SiO_2_ surface. This is because the hydrophilic cellulose could be potentially transformed into medical coatings on implant materials like polycaprolactone, as we demonstrated in our previous work [32]. For this purpose, we chosen PEI/CNCs multilayers prepared at 50 and 300 mM ionic strength where more adsorption of polymers were observed (Figure 3). The highest Δ*f*_3_ change was observed (45.93 ± 0.96 Hz) for the regenerated cellulose film compared to multilayers of PEI/CNCs, indicating high fibrin formation and deposition. This can be further supported by change in dissipation (Δ*D)*, which is lower for cellulose (*ΔD*_3_ = 6.2 ± 1.2 × 10^−6^) than for multilayers (*ΔD*_3_ > 10 × 10^–6^). It should be noted that the higher *ΔD*_3_ values reflect the formation of a less dense clot, a result of less fibrin formed and vice-versa [31]. The low anticoagulative activity of cellulose can be attributed to low surface charge and lack of anticoagulant functional groups (e.g., sulphate). To support that, CNCs, containing acidic sulfate ester groups (-OSO_3_^–^ H^+^), present in the top of bilayer 1 and 3 exhibited lower *Δf*_3_ changes than the cellulose (up to 8.5-fold). The bilayer 1 at 50 mM and 300 mM exhibited Δ*f*_3_ values of 10.8 ± 1.5 Hz and 8.6 ± 1.1 Hz, respectively, while the bilayer 3 at 50 mM and 300 mM showed *Δf*_3_ values of 6.4 ± 1 Hz and 4.2 ± 0.4 Hz, respectively. These observed lower Δ*f*_3,_ compared to cellulose, indicates that a low amount of fibrin was deposited on multilayers. Interestingly, the *Δf*_3_ obtained in this study for bilayer 1 was 2-fold lower than the value reported in our previous work for bilayer 1 prepared PEI/CNCs (the CNCs was isolated from microcrystalline cellulose) [15]. This was due to the higher density or amount of deposited CNCs in bilayer 1. The observed Δ*f*_3_ values for CNCs multilayers and neat cellulose are far lower than the values obtained for synthetic materials (poly(methyl methacrylate: −550 Hz; polyethylene terephthalate: −1400 Hz; polydimethylsiloxane:–750 Hz) [31,46,47] or sulfo-chitosan (–30 Hz) [47]. Furthermore, a significant difference in the final *ΔD*_3_ values between the multilayers and cellulose was observed indicating that the formed clot is less dense in the former case. The bilayer 1 at 50 mM and 300 mM exhibited *ΔD*_3_ values of 16.5 ± 2 × 10^–6^ and 14 ± 2 × 10^–6^, respectively, while the bilayer 3 at 50 mM and 300 mM exhibited *ΔD*_3_ values of 17 ± 2 × 10^–6^ and 18.03 ± 2.5 × 10^–6^, respectively. Figure 8D shows that the total coagulation time increased with number of bilayers and with ionic concentrations of NaCl compared to neat cellulose, which was in good correlation with the amount of deposited fibrin (see Figure 8C). For the positive control sample (cellulose film), a coagulation time of ca. 10 min e of 10 ± 2 min was observed. No major difference in coagulation time was observed for the bilayer 1 at 50 mM. A similar value was obtained for bilayer 1 at 300 mM. In contrast, the coagulation time observed for bilayer 3 at 300 mM NaCl was increased to ca. 14 min, which is ca. 4 min more than the positive control sample. However, the increase in coagulation time for bilayer 3 at 300 mM is still lower than the value (ca. 22 min) observed for the negative control sample i.e., heparin sulfate [46]. This could be due to the presence of higher density of sulfate groups in heparin. Thus, it can be concluded that the LBL assembly of CNCs suppressed fibrin formation and prolonged clotting time, thus exhibiting significant anticoagulant activity compared to the positive control neat cellulose [15,32], but less significantly than the negative control i.e., heparin sulfate.

**Figure 9:**
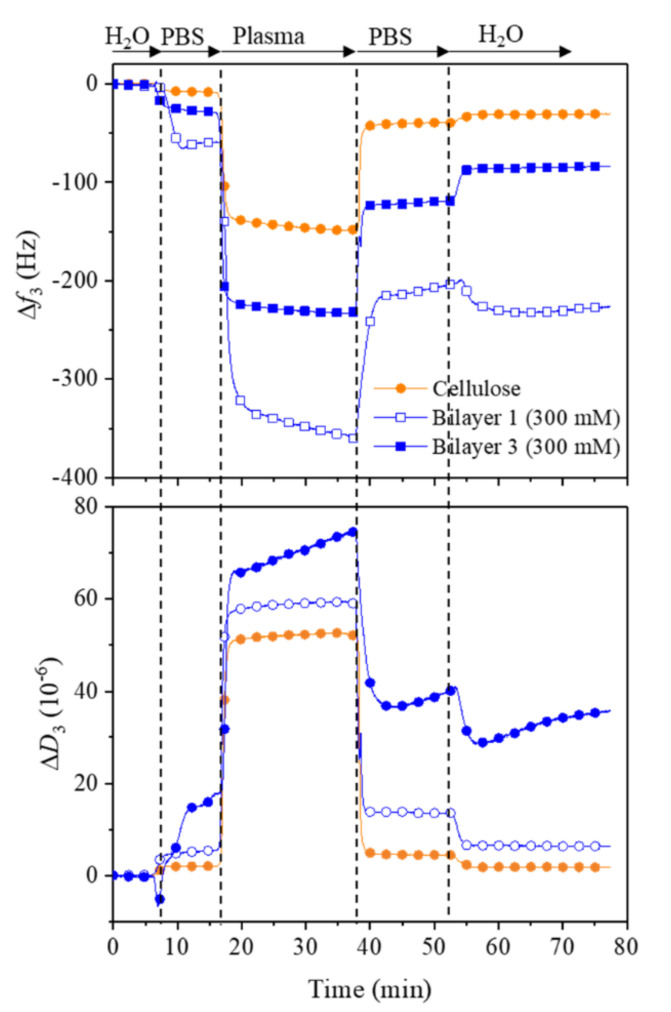
QCM-D change in frequency (**A**) and dissipation (**B**) for the adsorption plasma on bilayer 1 and 3 built on cellulose films.

To understand the adsorption of serum proteins and coagulation factors on our multilayers fabricated on cellulose, we carried out the adsorption of human plasma (100%) under physiological conditions (37 °C, pH 7.4) with QCM-D (see Figure 9). It should be noted that the human plasma used in this study contains different types of proteins and coagulation factors (according to the manufacture) that play an important role in preventing coagulation, and therefore they were chosen here to evaluate their adsorption characteristics on the above-mentioned surfaces. The introduction of plasma after water and PBS buffer showed a large decrease in frequency and an increase in dissipation for all samples. However, these changes in frequency (*Δf*_3_) and dissipation (*ΔD*_3_) were more pronounced for the bilayers compared to the controlled samples. After rinsing with PBS buffer and water, most of the adsorbed plasma was removed, as reflected by increased and decreased *Δf*_3_ and *ΔD*_3_. The final adsorbed mass of plasma is in the following order: Bilayer 1 (–165 ± 6 Hz) > Bilayer 3 (–55 ± 2 Hz) > Cellulose (–22 ± 3 Hz). It is suggested that the hydrophilic nature and strong hydration forces of the surfaces are responsible for the lower adsorption of the plasma on the multilayers. Since there are no studies published on the plasma adsorption of cellulose nanocrystals, we cannot compare our result with the literature data. It should be mentioned that the absorption of plasma refers to both platelet and protein adsorption. The content and concentration of proteins derived from platelet-poor plasma varies from to patient-to-patient. Of course, there are efforts to analyse the proteins composition in plasma, and a range of proteins have been determined [48,49]. However, in this work, QCM-D only determines the combined mass of both proteins and platelets derived from plasma and the unspecific or specific adsorption of the plasma to chosen surfaces.

## 4. Conclusions

In this study, we reported the preparation and multilayered assembly of cellulose nanocrystals on two types of hydrophilic substates: cellulose and SiO_2_ surfaces using layer-by-layer approach. The cellulose nanocrystals extracted from Isora plant fibers by sulfuric acid hydrolysis showed a length and diameter of 200–300 nm and 10–20 nm, respectively, and a negative zeta potential of–37 mV at pH 7.4. These monodispersed nanocrystals showed exceptional stability in water at varying concentrations and over extended period (3 weeks). The sequential assembly oppositely charged polyethyleneimine and cellulose nanocrystals investigated using QCM-D showed that more mass was adsorbed on cellulose surface than on SiO_2_ surface. Even though the adsorbed mass of both PEI and CNCs was increased with respect to increase of ionic strength of PEI solution, more mass deposited at higher ionic strength of NaCl. The deposited mass of polymers increased up to two bilayers compared to bilayer on both investigated surfaces. The fabricated multilayers with increasing ionic strength of PEI solution showed a uniform surface morphology, with increased surface roughness and layer thickness compared to neat cellulose and SiO_2_ surfaces. The multilayers of CNCs assembled on cellulose surfaces exhibited improved anticoagulant activity than the control cellulose sample. However, the plasma adsorption results showed that more plasma were deposited on bilayer compared bilayer 3 and the neat cellulose film.

## Figures and Tables

**Table 1:** Atomic force microscopy (AFM) root mean square (RMS) roughness values of the (first) polyethylenimine (PEI) in bilayer 1, and (final) CNCs coating in bilayer 3 created on cellulose and SiO_2_ surfaces at different ionic strength of NaCl.

	Cellulose				SiO_2_			
	0 mM	50 mM	100 mM	300 mM	0 mM	50 mM	100 mM	300 mM
PEI	0.9	6.5	6.1	6.7	0.4	1	1.1	1.1
CNC3	-	9.2	7.2	6.8	-	7.1	5.6	5.8

## Data Availability

The data presented in this study are available on request from the corresponding author.

## Supplementary Materials

The following are available online at https://www.mdpi.com/2073-4360/13/6/939/s1, Figure S1: AFM images of PEI coated on cellulose and SiO_2_ surface.
