# Supplementary material for: Anticoagulant Activity of Cellulose Nanocrystals from Isora Plant Fibers Assembled on Cellulose and SiO2 Substrates via a Layer-by-Layer Approach
Source: Polymers (Basel). 2021 Mar 18;13(6):939. doi: 10.3390/polym13060939 (PMC8003298; doi:10.3390/polym13060939)
Supplement: Supplementary file 1 [file polymers-13-00939-s001.pdf]

polymers MDPI

Supplementary materials: Anticoagulant Activity of Cellulose Nanocrystals from Isora Plant Fibers Assembled on Cellulose and SiO_2 Substrates via a Layer-by-Layer Approach

Tamilselvan Mohan, Cintil Jose Chirayil, Chandran Nagaraj, Matej Bracič, Tobias Alexander Steindorfer, Igor Krupa, Mariam Al Ali Al Maadeed, Rupert Kargl, Sabu Thomas and Karin Stana Kleinschek[2]

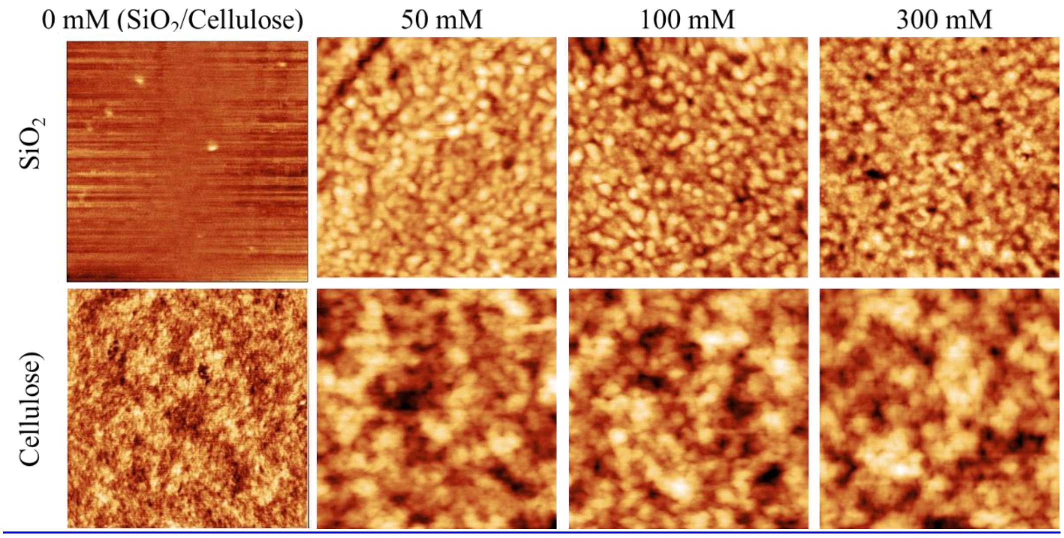

Figure S1. AFM heigh images of neat SiO_2 and cellulose surfaces coated with PEI layer at different ionic strengths of NaCl. Image size: 1 µm x 1µm. z-scale: 10 nm.
